# Effects of fish caudal fin sweep angle and kinematics on thrust production during low-speed thunniform swimming

**DOI:** 10.1242/bio.040626

**Published:** 2019-07-15

**Authors:** Alexander Matta, Javid Bayandor, Francine Battaglia, Hodjat Pendar

**Affiliations:** 1 CRashworthiness for Aerospace Structures and Hybrids (CRASH) Lab, Department of Mechanical and Aerospace Engineering, University at Buffalo, The State University of New York, Buffalo, NY 14260, USA; 2 Computational Research for Energy Systems and Transport (CREST) Lab, Department of Mechanical and Aerospace Engineering, University at Buffalo, The State University of New York, Buffalo, NY 14260, USA; 3 Department of Biomedical Engineering and Mechanics, Virginia Polytechnic Institute and State University, Blacksburg, VA 24061, USA

**Keywords:** Thunniform locomotion, Caudal fin, Thrust production, Fin sweep angle

## Abstract

Scombrid fish lunate caudal fins are characterized by a wide range of sweep angles. Scombrid that have small sweep-angle caudal fins move at higher swimming speeds, suggesting that smaller angles produce more thrust. Furthermore, scombrids occasionally use high angles of attack (AoA) suggesting this also has some thrust benefit. This work examined the hypothesis that a smaller sweep angle and higher AoA improved thrust in swimmers by experimentally analyzing a robophysical model. The robophysical model was tested in a water tunnel at speeds between 0.35 and 0.7 body lengths per second. Three swept caudal fins were analyzed at three different AoA, three different freestream velocities, and four different Strouhal numbers, for a total of 108 cases. Results demonstrated that the fin with the largest sweep angle of 50° resulted in lower thrust production than the 40° and 30° fins, especially at higher Strouhal numbers. Larger AoA up to 25° increased thrust production at the higher Strouhal numbers, but at lower Strouhal numbers, produced less thrust. Differences in thrust production due to fin sweep angle and AoA were attributed to the variation in spanwise flow and leading edge vortex dynamics.

## Abstract (precis)

**Summary:** The study examines the impact of scombrid fish caudal fin sweep angle and angle of attack on thrust production across a range of Strouhal numbers using a robophysical model.

## INTRODUCTION

Swimmers with fusiform bodies are considered by many to be the most economical swimmers, especially at higher Reynolds numbers (Borazjani and Sotiropoulos, 2010; Lighthill, 1970; Lindsey, 1978). The narrow tail of the fusiform body and the actuation of the tail, which is separated from the body, allows the caudal fin to oscillate with reduced inertial recoil. This reduction in inertial recoil is less energetically costly. Prior studies have shown inertial recoil is more prominent in other body types with thicker tails (Lighthill, 1970; Webb, 1992) resulting in increased anterior oscillation.

However, the fusiform bodies with long narrow peduncle will not shed as much energy into the wake as a thicker tail (Feilich and Lauder, 2015), limiting its ability to produce thrust. Particularly in thunniform locomotion, which only the caudal fin has a significant lateral undulation, most thrust is produced solely by the swept (commonly described as lunate) caudal fin by means of leading edge suction generation (Chopra, 1975; Karpouzian et al., 2006; Magnuson, 1978). It is also thought that the swept planform of a lunate caudal fin is hydrodynamically more efficient than other planforms (van Dam, 1987). Thus, the fusiform body shape, along with a long propulsive wavelength, minimize tail resistance while increasing caudal fin thrust potential. It has even been suggested that thunniform locomotion may be the most efficient mode of swimming at high speeds (Sfakiotakis et al., 1999).

The Scrombridae family has many species that utilize thunniform locomotion, most notably the *thunnus* genus. Scombrid fish possess a lunate caudal fin whose sweep angle varies greatly among species (approximately 25°–50° between species) (Magnuson, 1978). It was observed that scombrid species with lower sweep angles have higher swimming speeds, measured in body length per second, than those with larger sweep angles (Magnuson, 1978). It is also observed that the lymphatic circulatory system in some scombrids actively decreases the sweep angle of their dorsal and anal fins in fast maneuvers (Pavlov et al., 2017). All this evidence leads us to hypothesise that caudal fins in scombrids with lower sweep produce greater thrust when tail/fin kinematics (e.g. St number, angle of attack) are kept consistent between the different fin types.

A tuna-mimetic robophysical model with interchangeable fins was designed and constructed to test the hypothesis. Three different fins with sweep angles of 30°, 40° and 50° were used in the trials to span the range of sweep angles possessed by scombrid fish (approximately 25°–50°). The robot was tested in a circulating water tunnel that allowed for simulated swimming speed to be controlled. Also, the tethered nature of a water tunnel-based experimental setup allowed for thrust to be directly measured. Not only was caudal fin sweep angle examined, but also the Strouhal number (St), freestream velocity and angle of attack (AoA). AoA is defined as the angle between the incoming flow vector and the chord line of the caudal fin. The incoming flow vector is the vector sum between the forward motion of the fish and the lateral movement of the tail. AoA is another topic of particular interest in relation to tuna, which are members of the scombridae family. Tuna are thought to sometimes use AoA larger than what is traditionally considered efficient (typical static stall angle of hydrofoil is between 10°–15°). Based upon fin kinematics and swimming speed data of kawakawa tuna in the literature, AoA was calculated for five data sets yielding AoA ranging from 7° to 31° (Magnuson, 1978). The use of high AoA during thunniform swimming also may be due to increased thrust when other fin parameters are kept consistent and is a secondary hypothesis of this study. In order to test this secondary hypothesis that higher AoA increases thrust, three discrete AoA of 15°, 20° and 25° were tested.

Different aspects of fish swimming have been extensively studied by biologists and engineers using robots and simulations. The effects of individually varying caudal fin parameters (e.g., aspect ratio, flexibility, kinematics) as well as the effects when changes in parameters are coupled (e.g., shape, area, aspect ratio) have been previously examined experimentally (Alvarado and Youcef-Toumi, 2005; Esposito et al., 2012; Kopman and Porfiri, 2013; Low and Chong, 2010; Ziyu et al., 2016). In one study, a robot with a thick peduncle paired with a low aspect ratio caudal fin was compared against a robot with a thin peduncle paired with a high aspect ratio tail, and it was found that the thin peduncle paired with the high aspect ratio caudal fin could achieve higher speeds than its counterpart (Alvarado and Youcef-Toumi, 2005). The tails and fins were allowed to passively bend and thus there could be a great deal of variation in the tail kinematics between the two robots. Another study utilized several flexible foils representing the tail and caudal fin. These foils varied aspect ratio of the caudal fin, and also varied the peduncle thickness of the tail. They also inherently had different areas, and it was recognized that the use of flexible foils makes it hard to separate the effect of shape and kinematics on hydrodynamics as they are coupled in this type of system (Feilich and Lauder, 2015). Lastly in this study, there was no one shape that inherently performed better than the others. A third study was conducted where fin aspect ratio was examined and the results of the study indicate that the highest aspect ratio fin produced the most thrust despite having a smaller area than the other caudal fins used (Low and Chong, 2010). Similar to other studies, the caudal fin rotation is passive allowing for fin shape to also influence fin kinematics.

Other caudal-related experiments include one where spanwise articulation of the caudal fin as well as caudal stiffness is examined. It has been found that fin cupping produced the most thrust and that optimal fin stiffness was dependent on a variety of factors including frequency, flow speed and fin shape (Esposito et al., 2012). Another study presented a bio-inspired robotic fish and its control methodology. The study focused on control of the robot but included thrust measurements for three different passive caudal fins, one rectangular, one trapezoidal and one biomimetic (Kopman and Porfiri, 2013). The fins had varying aspect ratio and area. No clear conclusions could be made about differences in their performance nor did the authors try to discuss these results in detail.

Furthermore, different effects of the caudal fin geometry have been studied computationally. One study examined a variety of caudal fin shapes, including lunate, triangular, homocercal and rectangular, but focused on leading edge vortex (LEV) formation and fluid flow patterns rather than thrust forces (Borazjani and Daghooghi, 2013). Furthermore, the examined caudal fins had different aspect ratios. However, the study discussed the occurrence of leading edge vortex (LEV) formation and that fin sweep may have an impact on stabilization of the LEV. Another computational study examined the difference in efficiency and vortex structures between lunate and triangular fins. It was found that the use of the lunate caudal fin resulted in higher efficiencies at higher swimming speeds, while the triangular fin was more efficient at lower speeds (Xin and Wu, 2013). This was an optimization study and both the area and aspect ratio varied between the examined caudal fins.

Even though the hydromechanics of fusiform fish employing thunniform locomotion (characterized by high aspect ratio swept tails) are significantly different from fish with other body types and swimming kinematics, there have been surprisingly few experimental studies on the shape (and to a lesser extent motion) of their caudal fin. However, there is a lack of experimental investigations that isolate the effect of caudal fin geometric shape in not only thunniform locomotion, but also all caudal fin based locomotion. This is the first experimental study of its kind to examine the effect of fin sweep angle uncoupled from other parameters such as aspect ratio, flexibility and kinematics, and also provides new insight on the effect of AoA on thrust production.

## RESULTS

### Effects of fin AoA

Thrust values are presented as the difference between the static body drag and total axial force acting on the robot during operation. The thrust values were calculated by adding the measured body drag to the raw thrust values measured. The purpose is to demonstrate the thrust produced by the tail, thus removing the effect of the body. Drag on the body will be slightly different between oscillating and non-oscillating states, therefore, the body-drag values represent the body-drag contribution to the whole system.

**Fig. 1.**
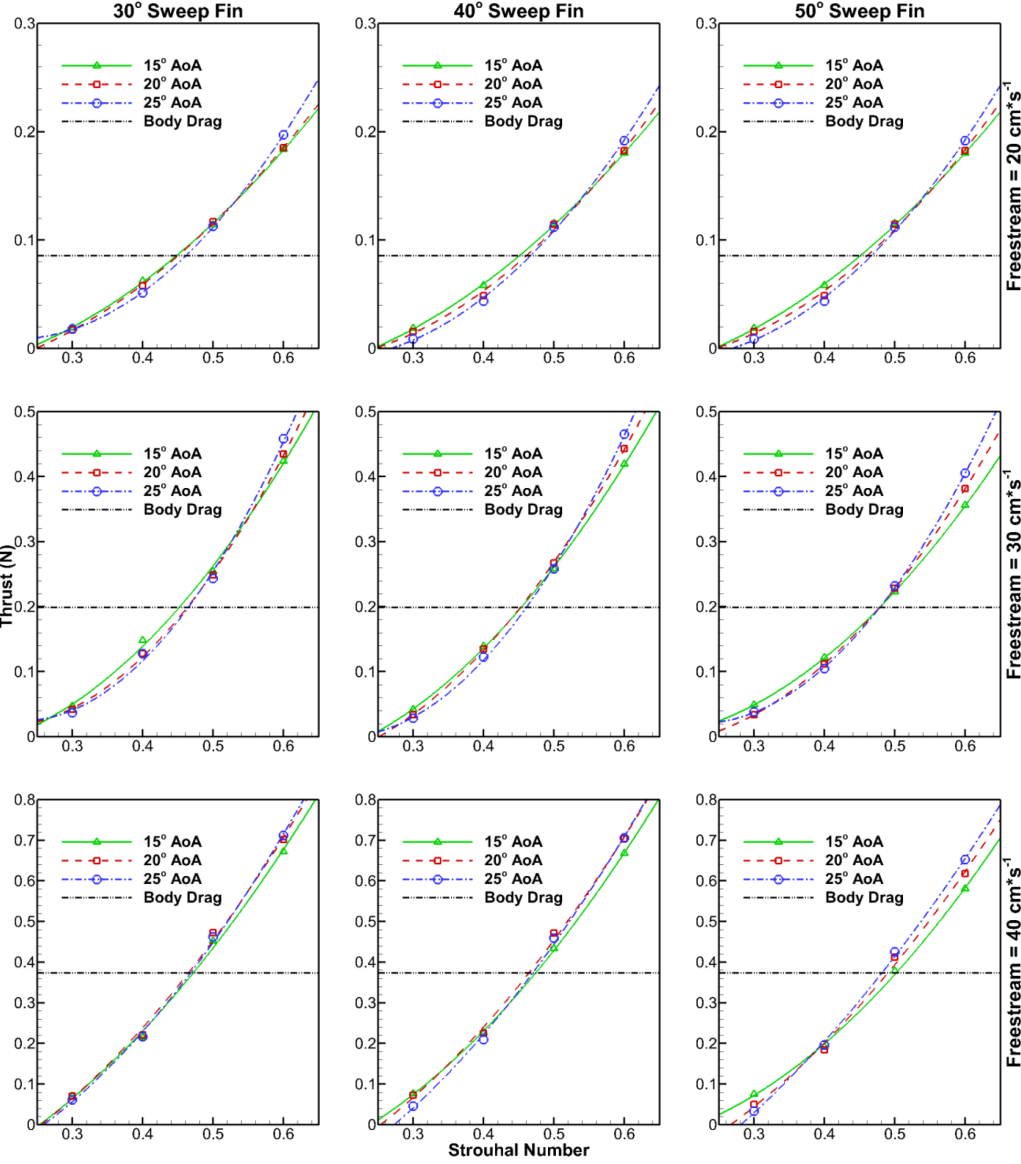
**Thrust production for all three fin types at different AoA.** Top row at 20 cm s^−1^ freestream, middle row at 30 cm s^−1^ freestream and bottom row at 40 cm s^−1^ freestream. Intersection with dashed body-drag line indicates St required to maintain constant swimming speed. Thrust values represent the measured body drag added to net system thrust.

There is a larger difference in thrust production between AoA at the highest St of 0.6 than at the lowest St of 0.3 (Fig. 1). At a St of 0.6, the largest AoA of 25° consistently produces the highest thrust, while the smallest AoA of 15° produces the lowest thrust. The relationship is reversed at a St of 0.3, where an AoA of 15° produces the most thrust for the majority but not all cases. Inconsistency of the results at a St of 0.3 may be due to random error which has more of an impact at lower St where thrust forces are smaller and fewer cycles were performed. The trend of higher AoA producing more thrust at the highest St holds true across all tested freestream velocities and fin sweep angles.

It can also be seen that the thrust curves always intersect the body-drag curves between St of 0.4 and 0.5. Again this intersection trend is present across all freestream velocities and fin sweep angles. The body-drag of the robot in its non-oscillating fully-extended state is given by the dashed line and increases with freestream velocity. At a freestream velocity of 20 cm s^−1^ the body drag is 0.086 N, at a freestream velocity of 30 cm s^−1^ the body drag is 0.20 N, and at a freestream velocity of 40 cm s^−1^ the body drag is 0.37 N.

### Effects of tail sweep angle

**Fig. 2.**
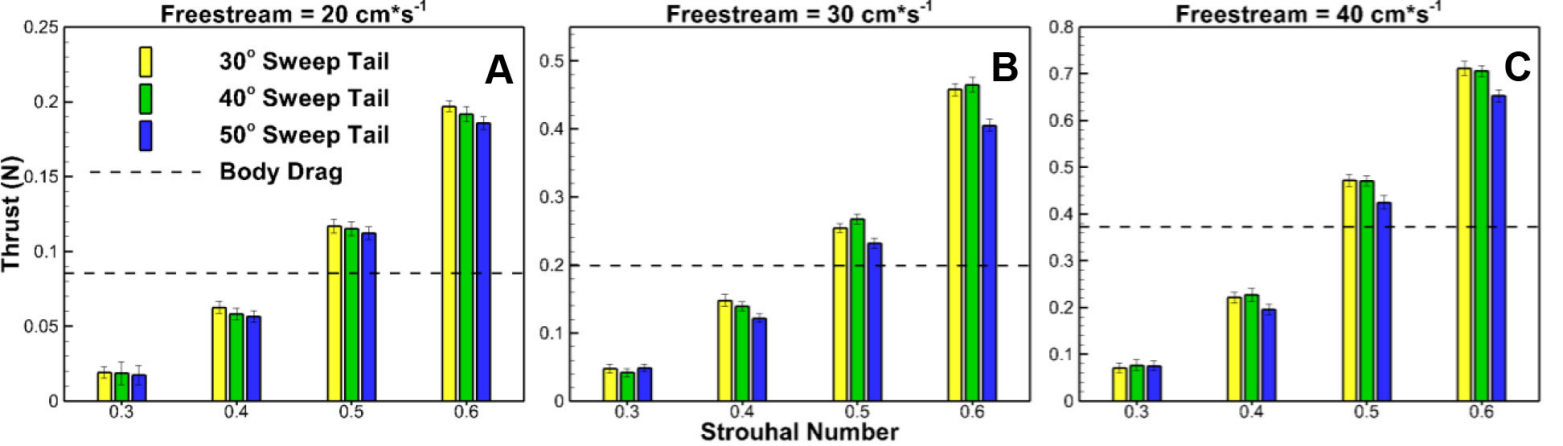
**Effect of caudal fin sweep angle on thrust.** (A) Thrust production of the three swept tails at a freestream velocity of 20 cm s^−1^. No significant difference in thrust production between tail types. (B) Thrust production of the three swept tails at a freestream velocity of 30 cm s^−1^. 50° swept tail produces significantly less thrust than the 30° and 40° swept tails at St of 0.4, 0.5 and 0.6. (C) Thrust production of the three swept tails at a freestream velocity of 40 cm s^−1^. 50° swept tail produces significantly less thrust than the 30° and 40° swept tails at St of 0.4, 0.5 and 0.6. Thrust values represent the measured body drag added to net system thrust.

Fig. 2A–C compares thrust production from different fin sweep angles at different St, and each subfigure corresponds to a different freestream velocity. For each St, the AoA used is associated with maximum thrust production: a 15° AoA is associated with St of 0.3 and 0.4, a 20° AoA is associated with a St of 0.5, and a 25° AoA is associated with a St of 0.6. Error bars representing 95% confidence intervals are shown. The calculated confidence intervals indicate a statically significant difference (*P*<0.05) for several of the cases outlined below.

At the lowest St of 0.3, there is no statistically significant difference between the amount of thrust produced between all fin sweeps with overlap occurring among all the confidence intervals. Unlike at a St of 0.3, at an St of 0.4 there is a statistical difference (*P*<0.05) where the fin with a 50° sweep produces less thrust at freestream velocities of 30 cm s^−1^ and 40 cm s^−1^. At a St of 0.5, fin sweep angle also has a statistically significant effect (*P*<0.05). At this St, the fin with the highest sweep angle of 50° produces less thrust at freestream velocities of 30 cm s^−1^ and 40 cm s^−1^. Lastly, the decreased thrust production of the fin with a 50° sweep angle becomes even more pronounced at the highest St of 0.6. At St of 0.6, the 50° swept fin produces significantly less thrust (*P*<0.05) than both the 30° swept and 40° swept fins at freestreams of 30 cm s^−1^ and 40 cm s^−1^, and also less thrust than the 30° swept fin at a freestream of 20 cm s^−1^. Differences in the two fins with 30° and 40° sweep angles are statistically insignificant for all cases as there is always overlap in the confidence intervals.

## DISCUSSION

The intersection of the thrust curves and body-drag curves represents constant speed swimming that hypothetically would occur in an untethered scenario. Before the data were processed, the intersection point between the body-drag line and thrust curve is where the raw thrust data equaled zero (indicating no net force in the forward or backward directions). The addition of the body drag to the processed values shifted the x-axis by the body-drag amount. Thus, the body-drag line can be treated as a representation of the system x-axis net force. In nature, constant speed swimming never truly occurs, as there are always slight deviations in velocity as the force varies throughout the tail stroke. However, the presentation of the thrust and body-drag data in Fig. 1 provides a good approximation. Therefore, where thrust is above the body-drag line acceleration would occur, and where thrust is below the body-drag line deceleration would occur. One interesting trend is that the intersections of the three AoA thrust curves generally seem to happen in unison rather than in pairs. Also, the intersection of the thrust curves always occurs close to the intersection point with the body-drag line (never more than a 0.1 St difference). This could mean that intensive control of AoA during steady-state swimming is unnecessary and caudal fin motion through passive flexing may be a reasonable solution for underwater vehicles using BCF propulsion. The intersection point between the thrust curves and body-drag line remain relatively constant across freestream velocities implying that a constant St is necessary for steady-state swimming of the robot and potentially actual tuna in this speed regime.

### In depth analysis of AoA's effect on thrust

**Fig. 3.**
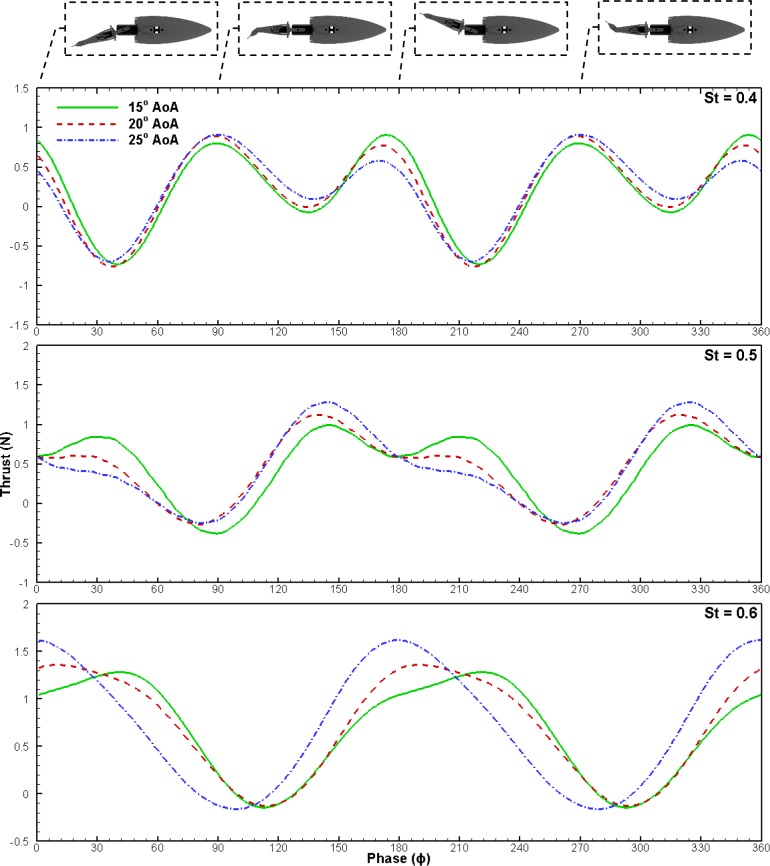
**Instantaneous thrust at three different AoA of 15°, 20° and 25°.** Data is from 30° sweep tail operating in 40 cm s^−1^ freestream. Stroke reversal points at ϕ = 0, 180 and 360. Thrust values represent the measured body drag added to net system thrust.

**Fig. 4.**
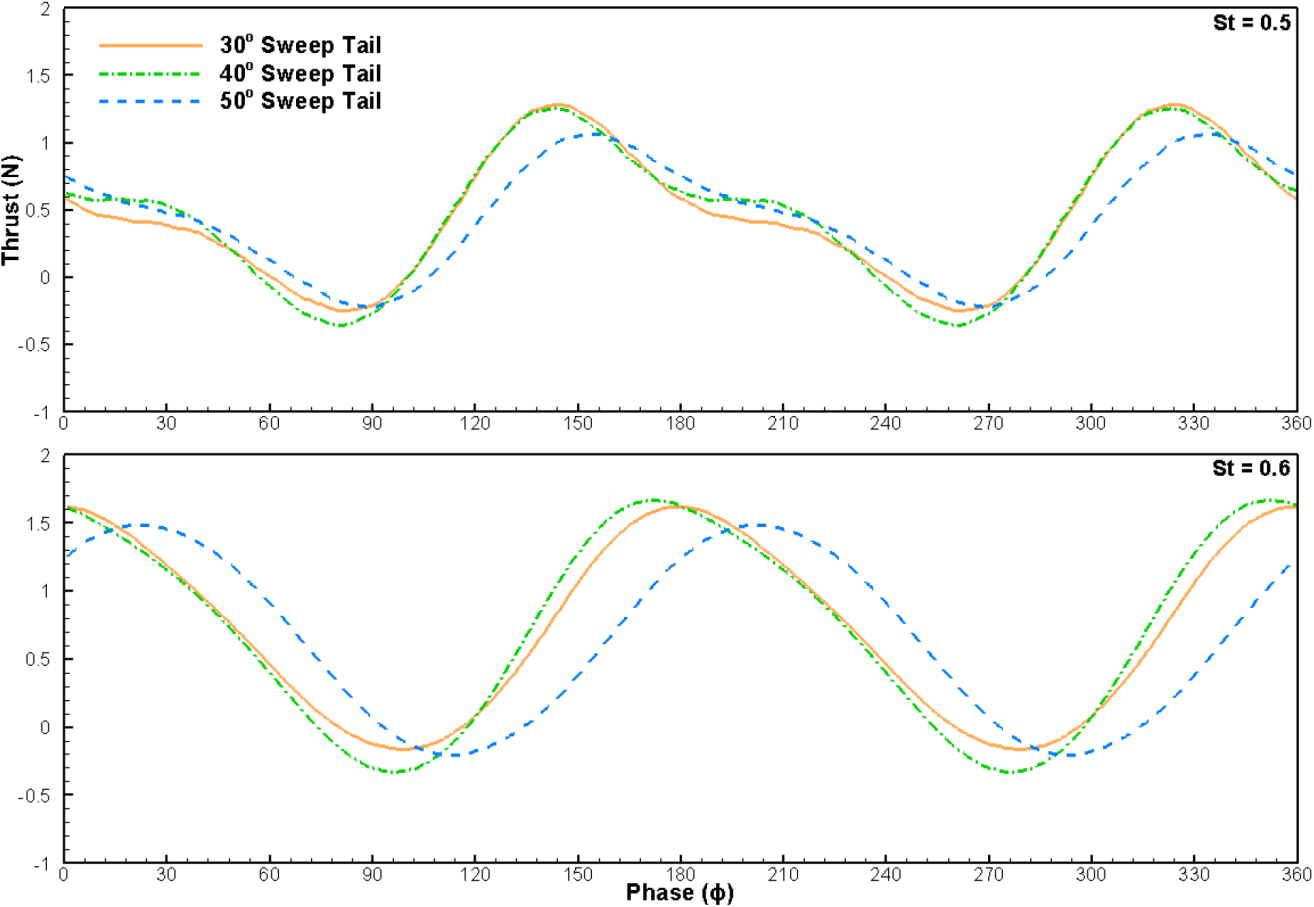
**Instantaneous thrust of the three swept tails operating with a 25° AoA in a 40 cm s^−1^ freestream.** Stroke reversal points at ϕ = 0, 180 and 360. Thrust values represent the measured body drag added to net system thrust.

The averaged thrust forces presented in the results section indicate that higher AoA produce increased thrust at higher St. In order to better understand why this trend occurs, phase-averaged instantaneous thrust at St of 0.4, 0.5 and 0.6 at a freestream velocity of 40 cm s^−1^ was examined (Fig. 3). It is important to note that these results do not isolate thrust force of the caudal fin from the rest of the tail. The results are thus a summation of forces in the streamwise direction from all tail structures. There are a variety of interwoven hydrodynamic effects at play including added mass, vortex development and traditional circulatory forces (used to characterize lift on aircraft wings), which makes it difficult to interpret the meaning of local maxima and minima of the trust force. These hydrodynamic phenomena seem to be out of phase with each other and thus the force time history drastically changes between St as the relative contribution of these phenomena increase or decrease. When examining instantaneous thrust, ϕ = 0° corresponds with the first stroke reversal point, ϕ = 90° corresponds with the first mid-stroke, ϕ = 180° corresponds with the second stroke reversal point, and lastly ϕ = 270° corresponds with the second mid-stroke (Fig. 3, Fig. 4). One full cycle (0°–360°) consists of two strokes and completes once the caudal fin returns to its original position.

**Fig. 5.**
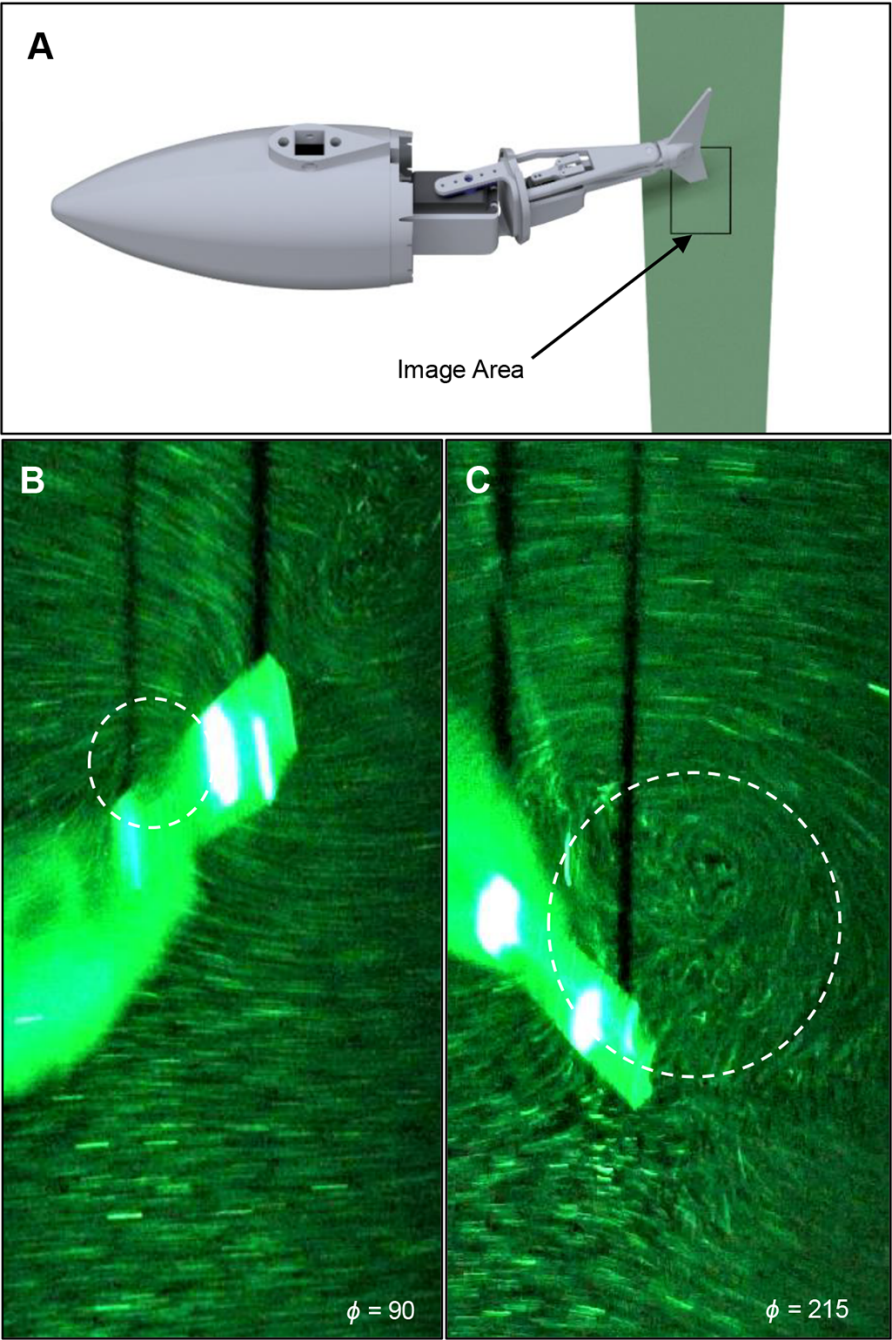
**LEV formation and destabilization.** (A) Laser sheet passing through the tail cross-section. Imaging plane is parallel to this sheet. (B) Newly formed LEV at ϕ = 90, (C) destabilized LEV ϕ = 215 on a 30° sweep fin. Visualization performed at a St of 0.5 and freestream of 30 cm s^−1^. Dashed circles show location of LEV.

However, one clear trend can be seen in Fig. 3. Higher AoA produces more thrust from the mid-stroke (ϕ = 90) to the stroke reversal (ϕ = 180), and lower thrust after the stroke reversal. Increased thrust between the mid-stroke and stroke reversal is expected as it is well known that higher AoA increases circulatory forces and LEV size. LEVs were expected to occur as all AoA are beyond that of the static stall angle of a thin oscillating rectangular cross-section (Kim et al., 2011). Flow visualization performed during several cases revealed LEV attachment, as seen in Fig. 5B, and subsequent destabilization and shedding shown in Fig. 5C, confirming LEV formation occurs.

Conversely, increased AoA causes a force hysteresis effect due to vortex shedding that likely results in the diminished thrust after the stroke reversal as demonstrated for flapping wings (Hubel and Tropea, 2009). Higher St may delay vortex shedding and the hysteresis effect for an oscillating caudal fin. For a basic plunging foil, it has been reported that the vorticity growth continued later into the cycle for each consecutively higher St (Eslam Panah et al., 2015). For the experiment presented here, the intersection point between the three AoAs shifts later in the cycle as St increases, which may suggest that vortex shedding is delayed. Furthermore, at higher St for a given AoA, the geometric angle of the fins’ normal vector is more in line with the freestream direction. The result is a larger component of the force caused by the low-pressure zone on the top surface of the fin in the thrust direction. Thus, the coupling of increased circulatory force and LEV, hysteresis delay and more optimal geometric angle are likely the cause of a higher AoA being more effective at higher St.

### In-depth analysis of sweep angle's effect on thrust

Another trend mentioned in the results section is the 30° and 40° sweep tails producing increased thrust over the 50° sweep tail. The thrust differences are not statistically significant for lower St of 0.3 and 0.4, but become more apparent for higher St of 0.5 and 0.6. Once again, in order to better understand the effect of fin sweep, phase-averaged instantaneous thrust at St of 0.5, and 0.6 and freestream velocity of 40 cm s^−1^ were examined, shown in Fig. 4. Very similar to the AoA comparison, change in fin sweep affects thrust during different parts of the stroke. The lower fin sweeps of 30° and 40° produce more thrust than the 50° sweep fin from the mid-stroke to the stroke reversal point, whereas the 50° sweep fin produces more thrust after the stroke reversal point.

In order to understand this trend, swept caudal fins can be compared with the swept wings found in aircraft, which are well understood. Wing sweep reduces the lift slope of the wing resulting in a lower lift coefficient for a given AoA, corresponding with lower thrust. Thus, smaller wing sweeps have a smaller effect and larger sweeps have a larger effect on lift coefficient. Theoretically this relationship is given by:(1)
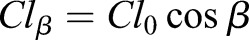
where *β* is wing-sweep angle, *Cl_o_* is the non-swept lift coefficient and *Cl_β_* is the lift coefficient of a swept wing (Katz and Plotkin, 2001).

An experimental study by (McArthur, 2008) confirms that this effect also occurs at the lower Reynolds numbers similar to those found in thunniform swimming. The higher lift coefficients of the 30° and 40° sweep fins relative to the 50° sweep fin are likely the reason they produce more thrust from ϕ ≈ 90 to 180. This region is where AoA is highest, the LEV has developed, and the fin is traveling fastest laterally.

However, fin sweep does not only have a detrimental effect on thrust production. Swept fins also produce more spanwise flow than non-swept fins, especially during the pitching of the tail at the stroke reversal point (Kim and Gharib, 2011). In three dimensions, it has been shown that vortex stability is linked to spanwise flow (Ellington et al., 1996; Maxworthy, 1981; Sane, 2003). This is because convection of the vorticity of the LEV towards the fin tip ensures that the LEV does not grow too rapidly and destabilize (Borazjani and Daghooghi, 2013; Ellington et al., 1996; Lentink and Dickinson, 2009; Maxworthy, 1981). Due to the increased spanwise flow, the LEV on the fin with 50° sweep is likely the most stable, resulting in the best performance during and after stroke reversal.

All of the tested fin sweep angles fall within a range of sweep angles that are characteristic of tuna (Magnuson, 1978). As tuna swim at various speeds and St, this variety of fin sweep angles is not surprising. However, for swimming at higher St, large sweep angles are not ideal for maximum thrust production.

### Future outlook

Increased thrust is likely not the only reason variation in caudal fin geometry and kinematics occurs in scombrids. Possible future studies include looking at lower AoA. Flow over fins with smaller AoA would likely be completely attached. As many of the differences between the fins and AoA seem to be related to vortex formation, vortex stability and flow reattachment, force time histories (and consequently averaged thrust) at lower AoA may be completely different. Furthermore, while LEV formation is known to significantly increase potential hydrodynamic and aerodynamic force, it is not necessarily the most efficient mode of operation making it worth investigating.

Also, it would be beneficial to look into not just thrust force but also lateral force as this gives an idea of the power required for fin motion as well as the degree of yaw and lateral displacement that would occur during free swimming. Regardless, this study suggests that in general, fin sweep has significant impact on swimming performance and warrants further study.

## CONCLUSION

The role of the caudal fin sweep on thunniform swimming was analyzed using a mimetic robot built for this study. The tested biomimetic robotic tuna showed differences in thrust production between the three caudal fins of different sweep angle and the three programmed AoA. Over the tested Reynolds number range, results indicated that the fin with the largest sweep angle of 50° resulted in lower thrust production than the 40° and 30° fins, especially at higher St, which is in agreement with the primary hypothesis. However, results showed less difference between 40° and 30° fins indicating that there are diminishing returns as fin sweep decreases.

Results also indicated that AoA up to 25° increased thrust production at the higher St, which is in agreement with the secondary hypothesis, but produced less thrust than lower AoA of 15° at the lower St. Additionally, results show that at St necessary for constant speed swimming, difference in AoA has less of an impact, suggesting that caudal fin angle may be more passively actuated during cruising swimming. Ultimately the relationship between AoA and thrust is complex and not independent of other fin parameters such as St.

Differences in thrust production due to change in fin sweep angle may have two causes. First, the magnitude of spanwise flow could change for different fin sweep angles helping stabilize LEVs. Second, attached flow lift coefficient may also vary with different sweep angles. Theoretically the impact of geometric sweep on attached flow lift coefficient (which contributes to thrust production during swimming) is not directly proportional to sweep angle. Instead it is related by a sinusoidal function, thus impact of sweep angle on lift coefficient increases as sweep angle increases.

Differences in thrust production due to AoA changes appear to be related to LEV formation and shedding which consistently occurred during the experiment. Higher AoA likely results in larger LEVs that increase thrust between the mid-stroke and stroke reversal but decrease thrust after the stroke reversal. Higher St may decrease the negative impact on thrust caused by vortex shedding.

This study has only scratched the surface of the impact of caudal fin shape. There are a variety of different scenarios (high speed versus low speed) and parameters (efficiency, agility and stability) in which different fin shapes may be optimal. As the caudal fin is the primary propulsor of many of our ocean's animals, continued study of caudal fin shape is relevant and can yield a great many more insights.

## MATERIALS AND METHODS

### Design of tuna-mimetic robot

The majority of the parts on the tuna-inspired robot were 3D printed on a Prusa I3 mk2 using fused filament fabrication. The structural parts were printed from polylactic acid (PLA), a relatively rigid thermoplastic when compared to other plastics like nylon and abs, while the flexible skin that covers the tail was made out of thermoplastic polyurethane (TPU). The front half of the robot was designed to be buoyant. In order to promote buoyancy in the robot's front half, the naturally porous 3D-printed nose piece was sealed using a rubber spray coating.

**Fig. 6.**
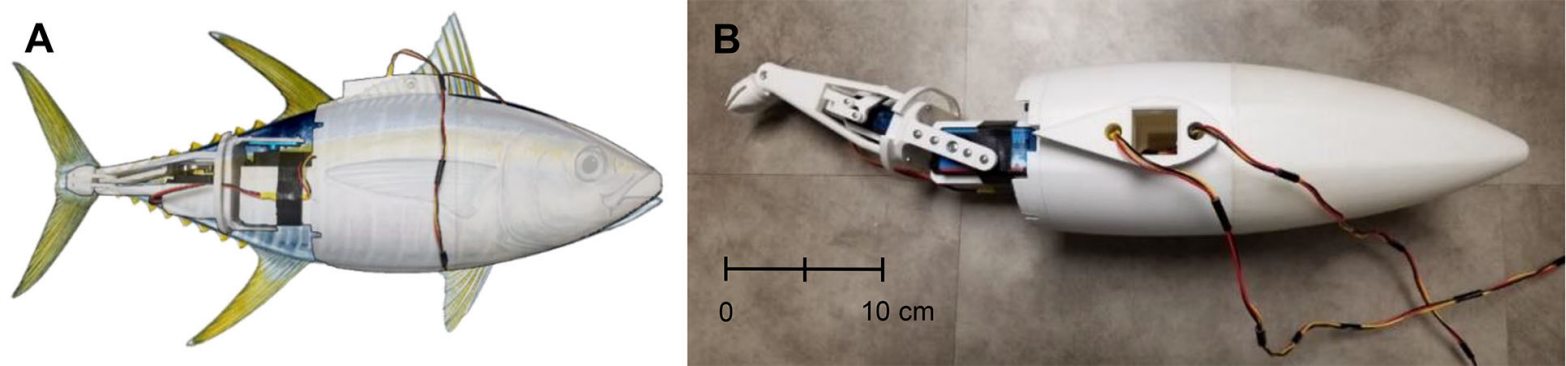
**Robot geometry.** (A) Side view of robot with tail skin removed, which adopts of the side profile of *Thunnus albacares*. Fork length of robot measures 57 cm. (B) Top view of robot with tail links deflected. Larger anterior servo attached directly to first tail pivot, smaller posterior servo controls second tail pivot via a four bar linkage.

The side profile of the tuna-mimetic robot was based on a yellowfin tuna, *Thunnus albacares* (Fig. 6). With a fork length (defined as the distance from the nose of a fish to the midpoint of the caudal fin trailing edge) equal to 57 cm, the robot was the size of a smaller yellowfin. Use of a smaller robot had several advantages including occupying less than 7% of the water tunnel cross-sectional area during the trials, thus reducing the impact of wall and blockage effects on thrust measurements. Furthermore, as the maximum freestream velocity of the tunnel was 0.5 m s^−1^, a higher body length (bl) per s swimming speed could be achieved due to the smaller size of the robot. Lastly, while yellowfin tuna can grow much larger than the robot, most of the kinematic data available are for tuna more comparable in size to the robot (Dewar and Graham, 1994).

Two independently controllable tail joints allow the tail motion of the robot to closely approximate that of thunniform swimmers. The two actuated joints also allow the interchange caudal fins to be subject to identical St. An independent actuator was used for each joint of the two tail joints. Both of these actuators were servos, shown in Fig. 6B. The larger actuator that controlled the tail base rotation was a Hitec D-845WP with a stall torque of 5.00 N m and a no-load speed of 58.8 rpm. The second smaller actuator controlled the fin angle and was a Savox SW-1210SG with a stall torque of 2.30 N m and a no-load speed of 76.92 rpm.

The pivot point of the larger actuator was located approximately one-third of the way between the tip of the peduncle and the nose of the robot. In order to control the caudal AoA separately from the body wave, the second pivot point was moved as close to the tip of the peduncle as possible without sacrificing structural integrity Fig. 6B. A linkage system was used to couple the actuator rotation to the peduncle rotation. This was done so an actuator that met the required toque and speed specifications could be used without increasing the width and height of the peduncle, an important geometric characteristic of tuna (Webb, 1992).

### Kinematics

Fine discretization of the body propulsive wave was deemed unnecessary, as the caudal fin is a larger contributor to thrust production than the rest of the body for the *Thunnus* genus (Dewar and Graham, 1994; Sfakiotakis et al., 1999). Since the chosen approach used actuators placed in series to discretize the fish tail movement, uncertainty in motion would be compounded with each additional actuator. Thus, using two actuators was appropriate as additional actuators would decrease reliability of motion and did not appreciably increase thrust.

In order to produce the tail and caudal fin motion, sinusoidal rotations were applied to the tail and fin links by the servos. It was assumed that the rotation of the caudal fin would have a 90° phase shift from its heaving motion, which is a reasonable assumption for heaving foils (Anderson et al., 1998). The servos were programmed to produce a tail sweep lateral displacement of 10 cm and a maximum AoA occurring at the mid-stroke (where lateral fin velocity is highest) of 15°, 20° or 25° in different trials. A 10 cm sweep displacement was chosen as it is 0.175 times the fork length of the robot, a value that falls within the range reported in the literature for live yellowfin tuna (Dewar and Graham, 1994). The AoA of 15°, 20° and 25° were chosen based on recommendations by (Anderson et al., 1998; Triantafyllou et al., 1993) stating that the range of optimal AoA for oscillating foils is approximately 15° to 25°.

### Caudal fin design

**Fig. 7.**
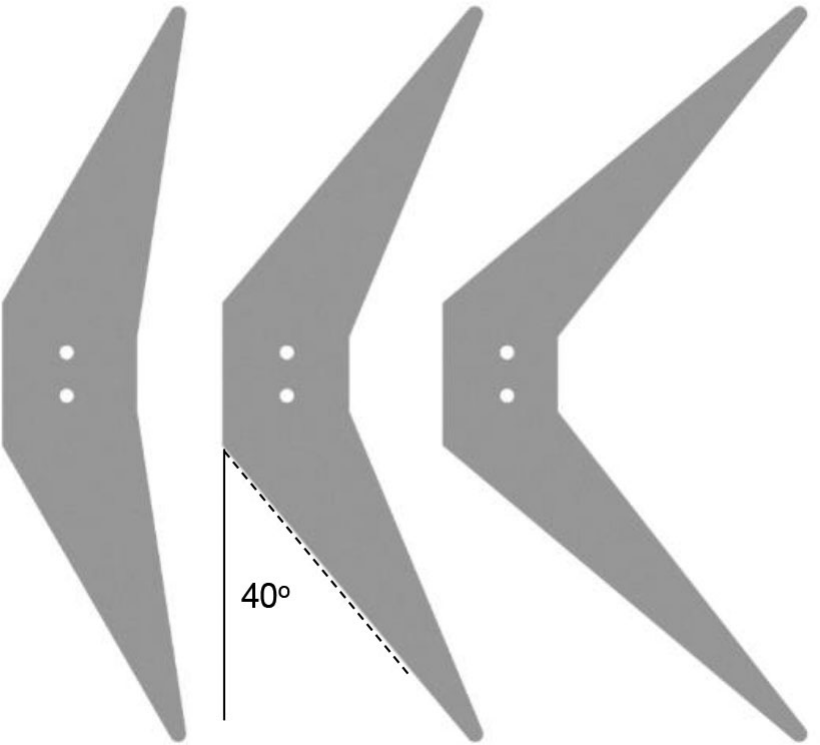
**Caudal fin shapes: from left to right, 30° sweep, 40° sweep and 50° sweep.** Laser cut from 3 mm acrylic and each having an identical surface area of 40 cm^2^ and aspect ratio of 7.2.

A total of three interchangeable caudal fins were used in the study (Fig. 7). Each fin is laser cut from clear acrylic to ensure accuracy and a smooth edge. Unlike many other experimental and computational studies, all of these fins have the same surface area of 40 cm^2^ and a tip-to-tip amplitude of 17 cm. The fin dimensions yield an aspect ratio of 7.2, which is comparable to a yellowfin tuna caudal fin aspect ratio, typically around 7 (Fierstine and Walters, 1968; Magnuson, 1978). The fins were designed to have sweep of 30°, 40° and 50° measured along the leading edge. For the tested motion and freestreams, the 3 mm acrylic fins where calculated to have negligible flex, and was an intentional choice as there is a coupling between shape and deformation, and deformation and hydrodynamics. By making the caudal fins sufficiently rigid, effect of shape rather than deformation on hydrodynamics could be isolated (Matta, 2017).

### Experimental setup

**Fig. 8.**
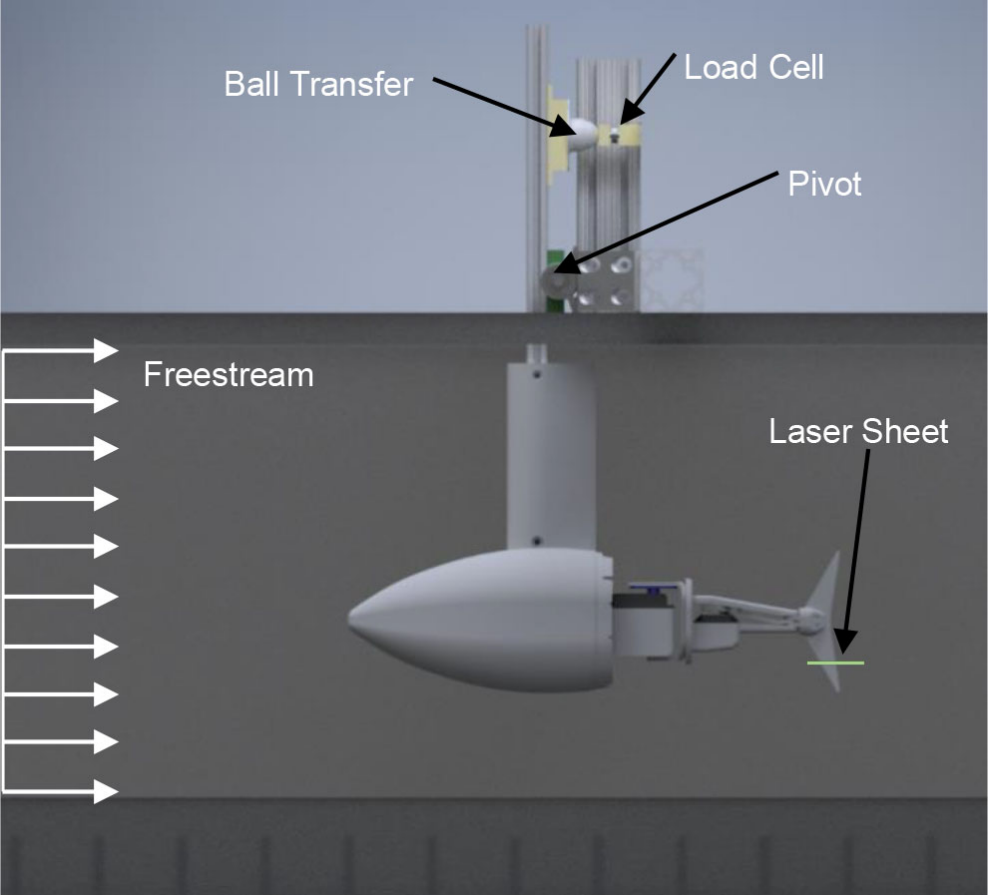
**Robotic prototype in a circulating water tunnel.** Robotic prototype transfers a mechanically-amplified thrust force to a load cell via a lever. Laser sheet intersects the caudal fin five-ninths of the way between the fork and tip.

The robot was tested in a water tunnel with a test section of 61 cm width and 61 cm height shown in Fig. 8. The experiment was run at three different freestreams of 20 cm s^−1^, 30 cm s^−1^ and 40 cm s^−1^, which equates to 0.35 bl s^−1^, 0.53 bl s^−1^ and 0.70 bl s^−1^. The dependent variable of interest was thrust production and was measured via a load cell (Omega LC201-25). The load cell was connected to an amplifier (Omega DMD4059) and the amplified signal was recorded with data acquisition (NI 9220 DAQ) with the sampling rate of 1 kHz. A lever with a low friction pivot was also used between the robot and the load cell to mechanically magnify the thrust force (Fig. 8). The mechanical amplification (3.9 for the experiment) is proportional to the ratio between the thrust axis to pivot point length and the load cell axis to pivot point length.

In order to reduce flow disturbances not present in nature, a streamlined sheath was placed around the pivot rod and a flexible skin around the tail linkages. The front section of the robot was sealed and filled with air, causing the center of buoyancy to always be in front of the center of gravity. A pitch-up configuration was preferred as it puts the load cell in adequate pre-compression without the use of springs or other mechanical devices that may cause damping. Pre-compression was necessary so a ball transfer attached to the lever rod always remained in contact with the interface block attached to the load cell. The ball transfer was used to ensure that a unidirectional force parallel to the load cell axis was applied to the load cell reducing the effects potential assembly misalignment and play (Matta et al., 2017).

### Data processing

Averaged thrust results were time-averaged from 30 s of data, collected after steady conditions had been reached. The time of 30 s includes a minimum of 18 cycles for the lowest Reynolds number and St cases, and a maximum of 72 cycles for the highest Reynolds number and St cases. Second-order polynomials were fitted to the time averaged thrust results (Fig. 1) as thrust, theoretically, should be proportional to the square of tail flow velocity (also proportional to St). Coefficients of determination for these curves are all above 0.99 indicating a good fit. Also, error bars were calculated for fin sweep average thrust results (Fig. 1). These error bars are indicative of a 95% confidence interval based on a t-distribution with each sample being the average thrust data of an individual cycle. Number for cycles associated with each freestream-St combination are shown in Table 1 below. All data processing was done using MATLAB and Tecplot 360.

**Table 1.**
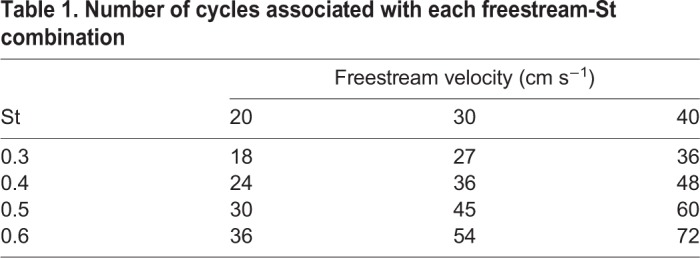
**Number of cycles associated with each freestream-St combination**

Furthermore, results were presented relative to body drag, meaning original net thrust measurements were shifted by body-drag measurements to provide only the caudal fin contribution to thrust. Body drag was recorded as the axial force on the robot in its non-oscillating neutral state (St = 0) when the water tunnel was operating at the specified freestream. Body drag acting on the robot was measured for each of the three tested freestreams.

### Preliminary flow visualization

A preliminary flow visualization was implemented to capture some of the large flow structures present around the fin. This flow visualization is not PIV and not intended to provide quantitative data but rather qualitative data to help guide the direction of future studies.

In order to perform flow visualization, the water tunnel was seeded with refractive microspheres and a laser sheet approximately 10 cm in width was projected normal to the caudal fin. This laser sheet intersected with the fin five-ninths of the way between the fin fork and the fin tip (Fig. 8). A camera (Sony α7R II) was used to capture images of the fin and surrounding area from below at a frame rate of 30 fps and a resolution of 3840×2160. A low shutter speed of 1/60 s was intentionally used to produce particle blurring yielding flow pathlines.
